# Genome-Wide Analysis of p53 Targets Reveals SCN2A as a Novel Player in p53-Induced Cell Arrest in HPV-Positive Cells

**DOI:** 10.3390/v16111725

**Published:** 2024-10-31

**Authors:** Yudi Zhang, Yi Liu, Xueyan Xing, Haibin Liu, Wuxiang Guan

**Affiliations:** 1 Center for Emerging Infectious Diseases, Wuhan Institute of Virology, Center for Biosafety Mega-Science, Chinese Academy of Sciences, Wuhan 430207, China; 2 University of Chinese Academy of Sciences, Beijing 100049, China; 3 Hubei Jiangxia Laboratory, Wuhan 430200, China; yiliu@wh.iov.cn (Y.L.);

**Keywords:** HPV, p53, SCN2A, p53 target, E6

## Abstract

The host transcription factor p53 is a critical tumor suppressor in HPV-induced carcinogenesis, regulating target genes involved in cell cycle arrest and apoptosis. However, the p53 targets have not been thoroughly analyzed in HPV-infected cells. In this study, p53 signaling in HPV16 and HPV18 cells was activated by depleting the viral oncoprotein E6. Subsequently, p53-regulated genes were identified by comparing them with genes altered in p53-silenced cells. True p53 targets were defined as genes with at least one overlapping p53 binding site and ChIP peak near their locus. Our analysis revealed that while some p53 targets were common to both the HPV16 and HPV18 cells, the majority of the targets differed between these two types, potentially contributing to the varying prevalence of HPV16 and HPV18 in cervical cancer. Additionally, we identified SCN2A as a novel p53 target involved in p53-induced cell cycle arrest in HPV-related carcinogenesis. This study provides new insights into the mechanisms by which p53 inhibits HPV-induced carcinogenesis.

## 1. Introduction

Persistent infections with high-risk human papillomaviruses (hrHPVs) are major causes of squamous epithelial cell carcinogenesis, particularly in the cervix [1,2,3,4]. The early proteins E6 and E7 of hrHPV are well-characterized oncoproteins essential for transforming virus-infected epithelial cells [5]. E7, by mediating the proteasomal degradation of retinoblastoma protein (pRb) and upregulating E2F transcription factors, plays a crucial role in immortalizing primary epithelial cells [6]. However, E7 alone cannot induce cellular immortalization [7], as it is accompanied by p53-induced apoptosis or senescence due to its abnormal replication stress [8,9]. Therefore, co-expression with E6, which directly binds and degrades p53 [10], is necessary for E7-expressing cells to escape p53-mediated anti-tumor responses during epithelial cell transformation [7,11].

p53, a tumor suppressor, senses genotoxic stress and triggers multiple pathways, including apoptosis, DNA repair, and senescence [12], to protect cells from tumorigenesis. It binds to a consensus motif containing two copies of 5′RRRCWWGYYY3′, regulating the genes involved in these pathways [13]. Normally, p53 is maintained at low levels, but its stability increases rapidly through post-translational modifications upon genotoxic stress, leading to p53 activation. In HPV-infected cells, p53 levels are very low, and the cells are resistant to p53-dependent anti-tumor pathways due to their constitutive E6 expression [14,15]. Thus, E6 is a promising target for reconstituting p53 pathways in HPV-induced carcinogenesis, and various E6-targeting approaches have been explored for developing effective anti-HPV therapies [16]. Silencing E6 with intron-specific small interfering RNAs (siRNAs) stabilizes and accumulates p53 in HPV16 and HPV18 positive cell lines, leading to efficient apoptotic cell death [8,17].

p53 exerts its tumor-suppressive functions through its target genes. For example, CDKN1A (p21), a well-characterized p53 target, inhibits all cyclin-dependent kinases and links DNA damage to cell cycle arrest following p53 activation [18,19]. However, p53 target genes regulated in HPV-infected cells are not well understood. In this study, we identified differentially expressed genes (DEGs) following p53 stabilization and knockdown in HPV16 and HPV18 positive cervical cancer cell lines. Given that p53 binding is consistent across different cell types and conditions [20], we combined p53 ChIP peaks from human foreskin keratinocytes (HFKs) [21] and predicted the p53 binding sites using JASPAR to profile the p53 target genes under HPV conditions. The genes regulated by both the E6 and p53, and containing overlapping p53 binding sites and ChIP peaks near their loci, were defined as p53 target genes. Finally, we identified both common and specific p53 targets in the HPV16 and HPV18 cells and demonstrated that SCN2A is a novel p53 target involved in p53-induced cell arrest.

## 2. Materials and Methods

### 2.1. Cell Cultures and Transfections

The CaSki and HeLa cells (ATCC) were maintained in Dulbecco’s Modified Eagle’s Medium (DMEM) (Thermo Fisher Scientific, Waltham, MA USA), supplemented with 10% fetal bovine serum (FBS) and 1% penicillin-streptomycin, and cultured at 37 °C with 5% CO_2_. SiRNAs targeting the HPV16 or HPV18 E6 were sourced from a previous report [17] and synthesized by Sangon Biotech Co., Ltd. Other siRNAs were designed and synthesized by Sangon Biotech Co., Ltd. (Table S4). The transfections were carried out using the TransIT-X2 Dynamic Delivery System (Mirus Bio, Beijing, China).

### 2.2. RT-qPCR

The gene expression was quantified using a SYBR Green qPCR assay. Two µg of total RNA was reverse transcribed to cDNA using HiScript II Q RT SuperMix for qPCR (#R222-01, Vazyme Biotech Co., Ltd., Nanjing, China). The qPCR was performed with Hieff^®^ qPCR SYBR Green Master Mix (#11201ES03, Yeasen Biotechnology Co., Ltd., Shanghai, China) using the primers listed in Table S4. The amplification efficiency ranged from 90% to 110%, with no dimers or nonspecific bands detected. The gene expression levels were calculated using the Comparative CT Method (ΔΔCT Method).

### 2.3. RNA-Seq and Data Analysis

The total RNA was extracted from the siRNA-transfected cells using TRIzol reagent (Thermo Fisher Scientific, #15596018). mRNA libraries were prepared and sequenced on the DNBSEQ platform (BGI-NGS-JK-RNA-001) as follows: mRNA was isolated using oligo (dT)-attached magnetic beads, then fragmented and quality-checked. cDNA synthesis was performed, with a single ‘A’ nucleotide added to the 3’ ends of the double-stranded cDNA for adaptor ligation. Following several cycles of PCR amplification, the products were denatured and cyclized, with the uncyclized DNA removed. Single-stranded circular DNA molecules were then amplified through rolling cycle amplification to generate DNA nanoballs containing multiple copies of DNA. These DNA nanoballs were loaded into patterned nanoarrays and sequenced using combinatorial Probe-Anchor Synthesis (cPAS).

The sequencing data were analyzed using the Tom Multi-omics Data Mining System (https://biosys.bgi.com, accessed on 3 September 2024). The raw reads were filtered with SOAPnuke to remove low-quality bases. The clean reads were mapped to the human genome hg38 using HISAT2. The gene expression levels were quantified using RSEM (v1.3.1). Differential expression analysis was conducted with DESeq2 (v1.4.5). KEGG enrichment analysis was performed using Phyper, based on the Hypergeometric test, with the significance levels of the terms and pathways corrected by the Q-value, applying a rigorous threshold (Q-value ≤ 0.05).

### 2.4. Genome-Wide Screening of p53 Targets

The genome-wide predicted binding sites of the p53 (MA0106.3) were downloaded from the JASPAR tracks in the UCSC Genome Browser (http://expdata.cmmt.ubc.ca/JASPAR/downloads/UCSC_tracks/2022/hg38/, accessed on 3 September 2024) and annotated using the ChIPseeker R/Bioconductor package. The p53 CHIP peaks were downloaded from the server of ReMap Atlas of regulatory regions (https://remap.univ-amu.fr/download_page, accessed on 3 September 2024), and the bed files were generated from a previous keratinocyte project (GEO accession number: GSE56674) [21]. The p53 binding sites overlapped with the p53 CHIP peaks were considered confirmed binding sites and then annotated using the ChIPseeker R/Bioconductor package. The bigwig files of H3k4me3 (https://hgdownload.soe.ucsc.edu/gbdb/hg38/bbi/wgEncodeReg/wgEncodeRegMarkH3k4me3/wgEncodeBroadHistoneNhekH3k4me3StdSig.bigWig, accessed on 3 September 2024) and H3k27ac (https://hgdownload.soe.ucsc.edu/gbdb/hg38/bbi/wgEncodeReg/wgEncodeRegMarkH3k27ac/wgEncodeBroadHistoneNhekH3k27acStdSig.bigWig, accessed on 3 September 2024) in normal human epidermal keratinocytes (NHEK) were from ENCODE Integrated Regulation tracks. The p53 CHIP peaks, binding sites, and H3k4me3 and H3k27ac marks were visualized in Integrative Genomics Viewer (IGV).

### 2.5. Cell Proliferation and Apoptosis Assays

The cell proliferation was measured by CCK-8. The CaSki cells (5000 cells/well) were seeded into a 96-well plate and transfected with siNS, siE6, siE6 and siSCN2A, or siE6 and siTP53I3 siRNAs, respectively. A total of 10 µl of CCK-8 reagent was added in each well and incubated for 1 h at 37 °C. The cell viability was then determined by the optical density (OD) at 450 nm.

The flow cytometric analysis was performed using the FITC Annexin V staining. The cell apoptosis was determined using a BD Pharmingen™ FITC Annexin V Apoptosis Detection Kit I. The CaSki cells (300,000 cells/well) were seeded into a 6-well plate and transfected with siNS, siE6, siE6 and siSCN2A, or siE6 and siTP53I3 siRNAs, respectively. The cells were incubated with FITC Annexin V and propidium iodide (PI) and then analyzed by flow cytometry at 72 h post-transfection.

## 3. Results

### 3.1. Knockdown of HPV Oncoprotein E6 Stabilizes p53 Protein and Alters p53 Signaling

**Figure 1:**
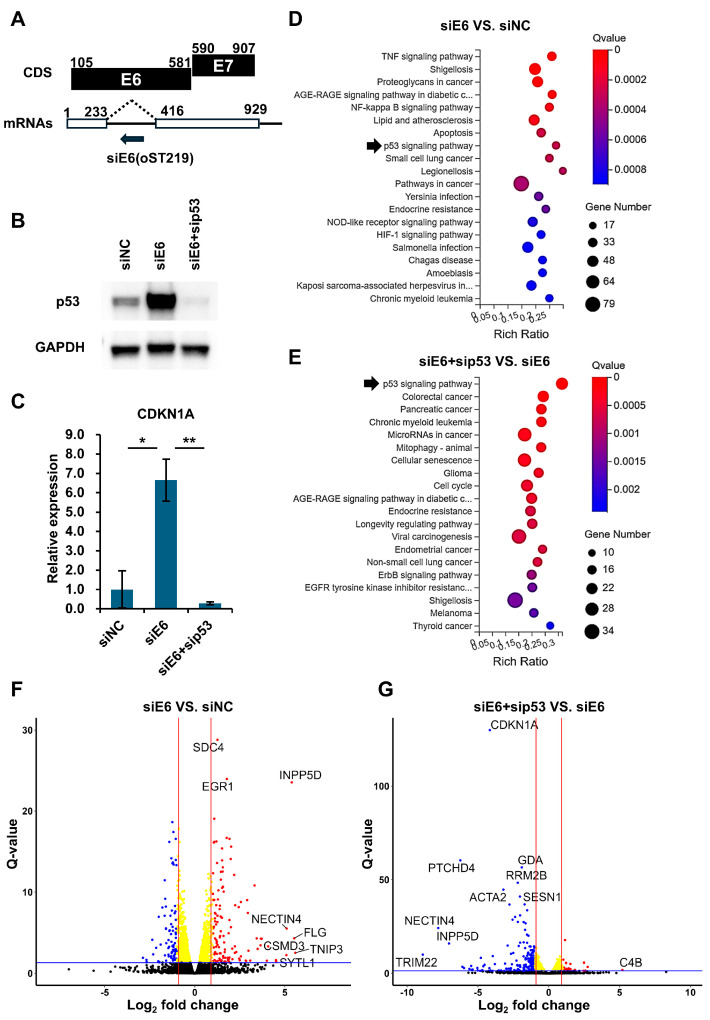
The knockdown of HPV oncoprotein E6 leads to p53 accumulation and gene regulation in the p53 pathway in the HPV18-positive cell line (HeLa). (**A**) Intron-specific siRNA (siE6) targets the un-spliced bicistronic RNA transcript, which contains both E6 and E7 ORFs but only expresses E6. E7 is expressed from the spliced transcript named E6*I. (**B**,**C**) HeLa cells were transfected with non-targeting siRNA (siNC), siE6, or siE6 + sip53. Western blotting (**B**) and qPCR analysis (**C**) show the protein expression of p53 and RNA expression of CDKN1A (p21), a p53-regulated gene, respectively. * *p* < 0.05; ** *p* < 0.01. (**D**,**E**) The KEGG pathway analysis of RNA-seq data from the siNC-, siE6-, or siE6- and sip53-transfected HeLa cells. The bubble charts display the top 20 enriched pathways in siE6 vs. siNC (**D**) and siE6 + sip53 vs. siE6 (**E**). p53 signaling pathways are highlighted by black arrows. (**F**,**G**) Volcano plots of the differentially expressed genes (DEGs) used for the pathway analysis, with the significant genes indicated.

To restore p53-mediated anti-tumor responses in the HPV-positive cells, the E6 expression was knocked down using E6 intron-specific siRNA (siE6), which does not affect E7 expression (Figure 1A and Figure S1A) [17]. This approach stabilized the p53 protein in both the HPV18 (HeLa) and HPV16 (CaSki) cells. The p53 and E6 knockdown were used to confirm the activation of p53 signaling. As expected, the p53 protein levels increased in the siE6-transfected cells and were subsequently depleted in the cells treated with both siE6 and sip53 (Figure 1B and Figure S1B). The major p53 target, CDKN1A, was regulated accordingly (Figure 1C and Figure S1C).

The RNA sequencing of three samples per knockdown condition generated over 40 million reads per sample. The average mapping ratio of the hg38 genome was above 95%, covering more than 16,000 genes (Table S1). The KEGG pathway analysis of the significant DEGs (Q-value < 0.05) in siE6 vs. siNC and in siE6 + sip53 vs. siE6 revealed that p53 signaling was one of the most altered pathways (Figure 1D,E and Figure S1D,E). Many of the most significantly upregulated genes in the siE6-transfected cells and downregulated genes in the siE6 + sip53-transfected cells were known p53 targets, such as CDKN1A, MDM2, and TRIM22 (Figure 1F,G and Figure S1F,G) [18,22,23,24,25]. These results suggest that the knockdown of HPV oncoprotein E6 not only stabilizes p53 but also alters gene expression within the p53 pathway.

### 3.2. p53 Functions More as an Activator than a Repressor in HPV-Positive Cell Lines

**Figure 2:**
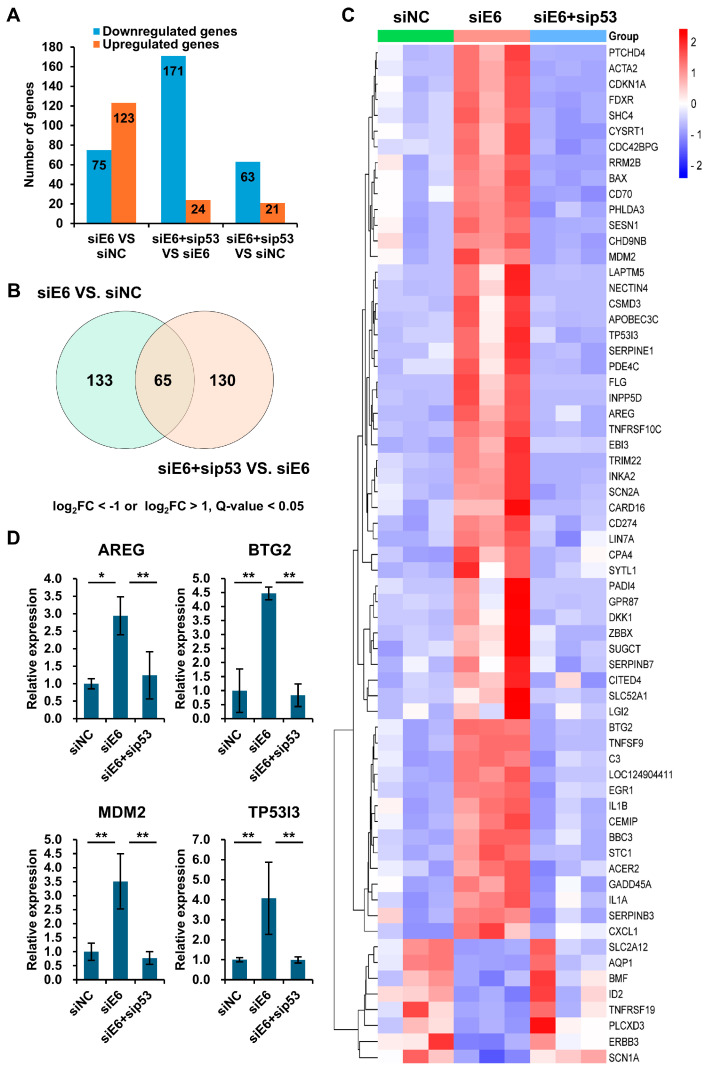
p53 functions as an activator in the HPV18-positive cell line (HeLa). (**A**) Bar graph showing number of DEGs (log_2_FC < −1 or log_2_FC > 1 and Q-value < 0.05) in siE6 vs. siNC, siE6 + sip53 vs. siE6, and siE6 + sip53 vs. siNC groups. (**B**,**C**) Venn diagram (**B**) and heatmap (**C**) of overlapping DEGs between siE6 vs. siNC and siE6 + sip53 vs. siE6 groups. (**D**) Validation of the expression of four DEGs in siNC-, siE6-, or siE6 + sip53-transfected HPV18 cells by qPCR. * *p* < 0.05; ** *p* < 0.01.

To identify p53 target genes from the RNA-seq data, we selected genes based on the significant DEGs (Q-value < 0.05) with a log_2_ fold change cutoff of ≥1 for the upregulated and ≤−1 for downregulated genes (Figure 2A,B and Figure S2A,B). Notably, there were more upregulated genes when the p53 was stabilized (siE6 vs. siNC), while more genes were downregulated when the p53 was knocked down (siE6 + sip53 vs. siNC or siE6 + sip53 vs. siE6) in both the HPV18 and HPV16 cells (Figure 2A and Figure S2A), indicating that p53 acts more as an activator than a repressor. The DEGs common to both the p53 stabilization and knockdown conditions were considered potential p53 target genes. A total of 65 and 112 DEGs were identified in the HPV18 and HPV16 cells, respectively (Figure 2B and Figure S2B). Remarkably, all these genes were oppositely regulated in the p53 stabilization and knockdown conditions, with 80% of them activated when the p53 was stabilized by the E6 knockdown (Figure 2C and Figure S2C). Some altered gene expressions were further verified by qPCR assays (Figure 2D and Figure S2D). Thus, the RNA-seq profiling reveals that p53 primarily functions as an activator for most DEGs in HPV-positive cells.

### 3.3. Identification and Characterization of p53 Target Genes by Annotation of p53 Binding Sites Around the Gene Locus

**Figure 3:**
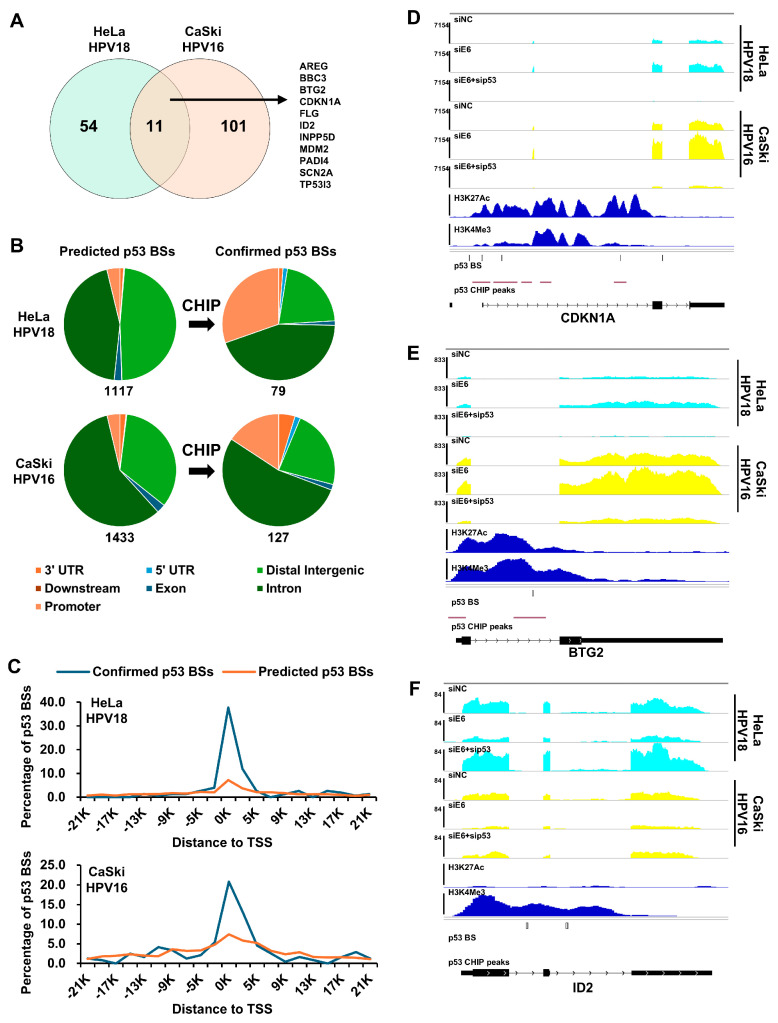
Identification and characterization of p53 target genes in HPV18 and HPV16 positive cell lines. (**A**) Venn diagram showing overlapping DEGs regulated by p53 in HPV18 (65 DEGs) and HPV16 (112 DEGs) positive cell lines, with gene symbols of 11 overlapped DEGs indicated. (**B**) p53 binding sites around DEGs were predicted using consensus p53 motif (MA0106.3) (left panel) and further confirmed by intersecting with p53 CHIP peaks from human foreskin keratinocytes (right panel). Pie charts show the proportion of annotated binding motifs. (**C**) Distribution of distances from predicted and confirmed p53 binding sites to nearest transcription start sites (TSSs). (**D**–**F**) Visualization of expressions, H3K27Ac mark, H3K4Me3 mark, p53 binding sites, and p53 CHIP peaks for CDKN1A (**D**), BTG2 (**E**), and ID2 (**F**) in Integrative Genomics Viewer (IGV). The top six tracks show read coverage in siNC-, siE6-, or siE6 + sip53-transfected HPV18 or HPV16 cells, with H3K27Ac and H3K4Me3 marks from normal human epidermal keratinocytes (NHEK), p53 binding sites, and p53 CHIP peaks shown below. BS: binding site.

We anticipated identifying many common p53 targets in both the HPV18 and HPV16 cells. However, only 11 common genes were found among the 65 and 112 candidates (Figure 2B, Figure 3A and Figure S2B). Most of these 11 genes are well-known p53 targets, such as BBC3, CDKN1A, and MDM2 (Figure 3A).

To determine if the candidates are directly targeted by p53, we annotated their p53 binding sites around their gene loci by combining the predicted binding sites and p53 ChIP peaks. Using the p53 motif matrix profile MA0106.3 from JASPAR, we identified 1117 and 1433 predicted p53 binding sites around the gene loci of the 65 and 112 candidates in the HPV18 and HPV16 cells, respectively (Figure 2B and Figure S2B and Table S2). Most of the binding sites were located in the intergenic or intron regions (Figure 3B, left panel, and Table S2). Given that p53 binding is consistent across different cell types [20], we used p53 ChIP peaks from HFK cells for further validation [21]. Only a few predicted binding sites were located within the p53 ChIP peaks and were considered confirmed p53 binding sites (Figure 2B, right panel, and Table S3). Compared to the predicted sites, many of the confirmed sites were found in the promoter region (Figure 3B), with 50% located within 3 kb of the transcription start sites (Figure 3C). For the HPV18 cells, 41 genes from the 65 candidates, which contained at least one confirmed binding site (Figure 2B and Table S3), were defined as true p53 target genes. Notably, p53 activated almost all these targets, except SLC2A12 in the HPV18 cells (Figure 2C and Table S3). In the HPV16 cells, 64 out of 112 candidate p53 targets were confirmed by the same method, with only four genes repressed by p53 (Figure S2B,C and Table S3). The common p53 targets in the HPV18 and HPV16 cells included AREG, BBC3, BTG2, CDKN1A, INPP5D, MDM2, PADI4, and SCN2A.

Gene expression correlations, active histone marks (H3K27Ac and H3K4Me3), p53 binding sites, and p53 ChIP peaks were visualized using IGV. The p53-repressed gene ID2, which did not contain p53 ChIP peaks, was also shown (Figure 3D–F and Figure S3A–G). These data illustrate how p53 recognizes specific DNA regions for gene transactivation. For instance, the p53 bound to both the promoter and gene body through multiple binding sites to regulate the CDKN1A expression (Figure 2D). In addition, qPCR analysis for ID2 expression was conducted. While the changes were consistent with the RNA-seq data, they were not statistically significant (Figure S3H). Upon re-examining the RNA-seq data, we noted that the variability among the down-regulated genes was much higher (Figure 2C bottom). This might be because these genes are not directly targeted by p53; for instance, no p53 ChIP peaks were found in the ID2 gene body (Figure 3F). Overall, we demonstrated that p53 activates the expression of various downstream targets by binding to their gene loci in HPV18 and HPV16 cells.

### 3.4. SCN2A Is Involved in p53-Induced Cell Arrest

**Figure 4:**
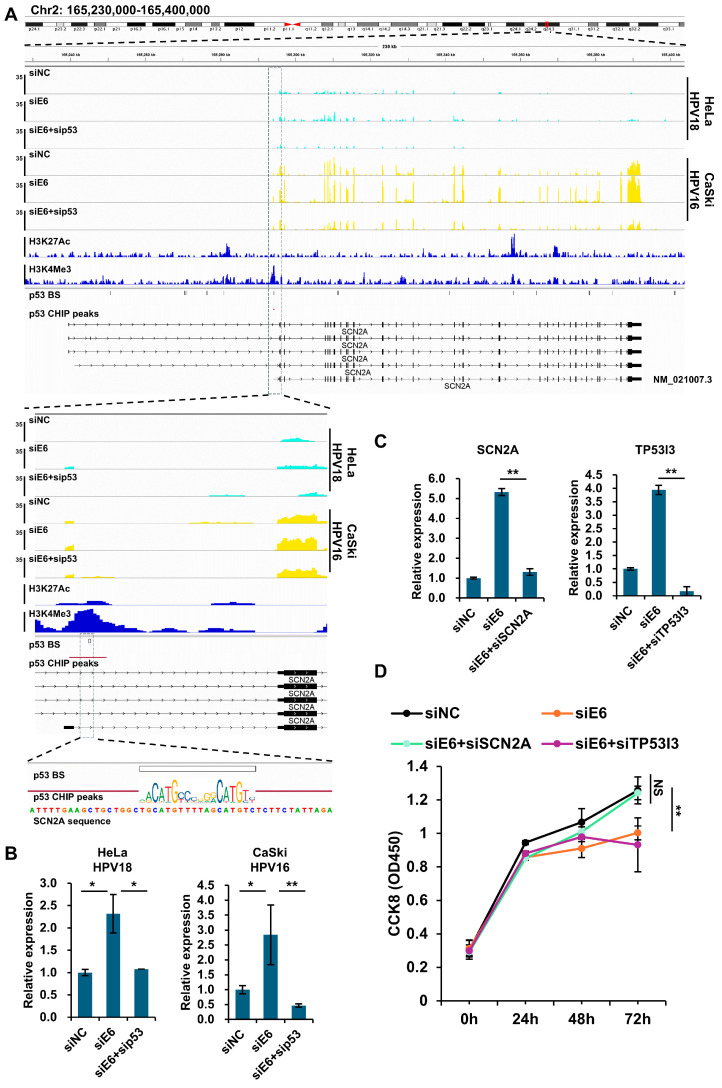
SCN2A is a common p53 target gene in both HPV18 and HPV16 positive cell lines and is involved in p53-induced cell arrest. (**A**) p53 activates the expression of the SCN2A transcript NM_021007.3 by binding to a p53 binding site near the TSS, which overlaps with the p53 CHIP peaks and H3K4Me3 mark. The genomic position of SCN2A on chromosome 2 and its expression, H3K27Ac mark, H3K4Me3 mark, p53 binding sites, and p53 CHIP peaks are shown by IGV. BS: binding site. (**B**) The validation of SCN2A expression in the siNC-, siE6-, or siE6 + sip53-transfected HPV18 or HPV16 cells by qPCR. (**C**) The knockdown efficiencies of SCN2A and TP53I3 in the siE6 + siSCN2A (**left**) and siE6 + siTP53I3 (**right**) cells (CaSki) were validated by qPCR. (**D**) The effects of E6, E6 + SCN2A, or E6 + TP53I3 knockdown on CaSki cell proliferation, measured by a CCK8 assay at the indicated time points post-transfection, with each point representing an average of six replicates. NS, not significant; * *p* < 0.05; ** *p* < 0.01.

SCN2A has not previously been characterized as a p53 target. This gene, located on chromosome 2 (q24.3), can be transcribed into at least five RNA isoforms, all encoding the voltage-gated sodium channel protein NaV1.2. According to the read coverage, only the RNA isoform NM_021007.3 was transcribed in the HPV cells (Figure 4A). A specific p53 binding site in the promoter region, overlapping with a p53 ChIP peak and an active H3K4Me3 mark, was responsible for this RNA transcription (Figure 4A). The p53-dependent expression was further validated by qPCR assays (Figure 4B). We silenced the SCN2A expression in E6-depleted CaSki cells to investigate its role in p53-induced apoptosis or cell arrest (Figure 4C), alongside another p53-regulated gene, TP53I3 (Figure 3A and Figure S3F). The SCN2A and TP53I3 expressions were significantly reduced in both the siE6- and siSCN2A-transfected cells compared to the siE6-transfected cells (Figure 4C). The E6-depleted cells and E6 + TP53I3-depleted cells showed a significant growth delay compared to the control cells, whereas the E6 + SCN2A-depleted cells did not (Figure 4D). This indicates that p53-activated SCN2A expression is crucial for p53-induced cell arrest in E6-depleted cells. We conducted a similar experiment in HeLa cells (Figure S4A). While the cells transfected with siE6 + siSCN2A grew faster than those with only siE6 transfection, the differences were not as significant as that in the CaSki cells. This might be because the siE6 had a lesser effect on the cell viability in the HeLa than that in the CaSki cells. However, the SCN2A was not involved in the p53-mediated apoptosis (Figure S4B).

### 3.5. p53 Activated Specific Targets Involved in Metabolism in HPV16 Cells

**Figure 5:**
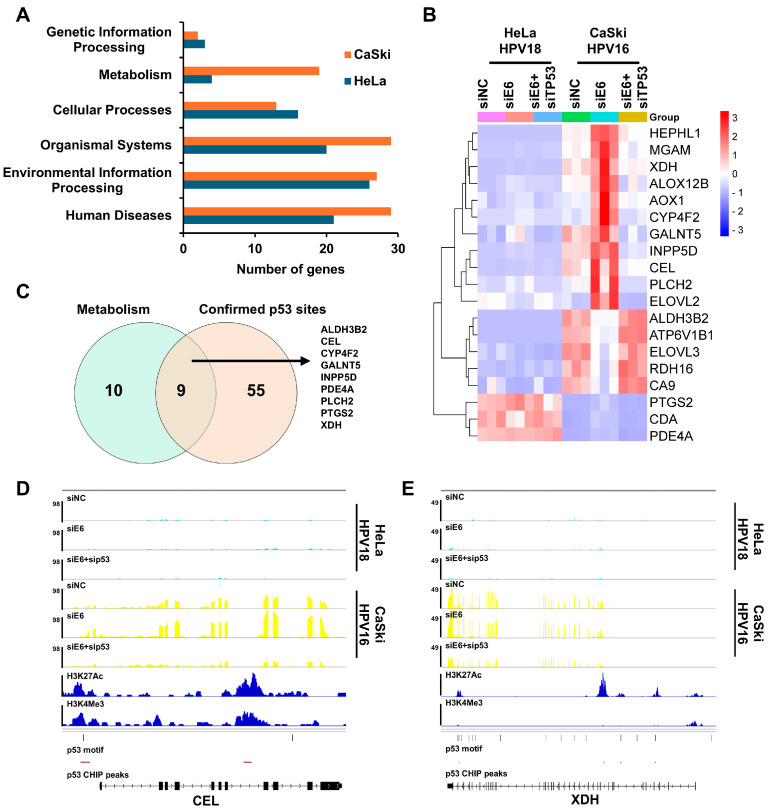
The identification of specific p53 target genes in the HPV16 positive cell lines. (**A**) Enriched pathways in the HPV18 and HPV16 positive cell lines, categorized based on the KEGG PATHWAY database, with a bar graph showing the number of gene hits in each category. (**B**) A heatmap showing the expression of gene hits in metabolism pathways from the HPV16 cells across all the samples. (**C**) The intersection of metabolism gene hits with confident p53 target genes in the HPV16 cells that contained confirmed p53 binding sites. (**D**,**E**) The visualization of the expressions, H3K27Ac mark, H3K4Me3 mark, p53 binding sites, and p53 CHIP peaks of the HPV16-specific p53 target genes CEL (**D**) and XDH (**E**) in IGV.

We demonstrated that the activated p53 targets differed between the HPV18 and HPV16 cells (Figure 3 and Table S3). To explore whether these targets have distinct biological functions, we categorized the most enriched KEGG pathways of the DEGs regulated in both the siE6 and siE6 + sip53 groups into various types and sub-types based on KEGG pathway maps (https://www.kegg.jp/kegg/pathway.html, accessed on 3 September 2024). The most differentially regulated biological function was metabolism (Figure 5A and Figure S5). In the HPV18 cells, only a few genes were enriched in metabolism (Figure S5). Genes enriched in metabolism in the HPV16 cells were either not expressed or not regulated by p53 in the HPV18 cells (Figure 5B). Among these genes, ALDH3B2, CEL, CYP4F2, GALNT5, INPP5D, PDE4A, PLCH2, PTGS2, and XDH were further defined as p53 targets because their gene loci contained at least one confirmed p53 binding site, by overlapping with 64 p53 targets containing 127 confirmed p53 binding sites in the HPV16 cells (Figure 5C and Table S3). For example, CEL expression was only regulated by p53 in the HPV16 cells and had a confirmed p53 binding site in its promoter region (Figure 5D), while XDH had three confirmed p53 binding sites in its gene body (Figure 5E). Thus, p53 regulates cell metabolism through specific targets in HPV16 cells.

## 4. Discussion

p53 is a well-characterized tumor suppressor that regulates numerous targets in response to cellular stress, controlling cell fate [12,26]. Elevated p53 levels during stress lead to the widespread binding of p53 to DNA, influencing the transcription of genes involved in cell cycle arrest and apoptosis. In HPV-infected cells, however, p53 is degraded by the oncoprotein E6, which blocks the p53 signaling pathways crucial for cell transformation and tumorigenesis. Reconstituting the p53 pathway, particularly targeting key downstream genes, presents a promising strategy for managing HPV-infected cells and altering cell fate. In this study, we mapped p53 binding sites and ChIP peaks around p53-regulated genes in HPV cell lines, identifying distinct p53 targets in HPV16 and HPV18 infections. Notably, SCN2A emerged as a novel p53 target important for inducing cell arrest.

Silencing E6 with siRNAs has been shown to stabilize p53 protein in HPV-infected cells, resulting in reduced cell growth, colony formation, and increased apoptosis [8,17,27,28]. This treatment also enhances sensitivity to radiotherapy, demonstrating a synergistic antitumor effect [29]. However, the specific p53 targets activated by E6 silencing had not been elucidated. Since E7 and other viral factors interact with p53 targets [30,31], the p53 targets activated by E6 silencing might differ from those affected by other cellular stresses. In this study, p53 signaling was significantly altered by E6 knockdown in both the HPV16 and HPV18 cells (Figure 1D and Figure S1D), and p53-regulated genes were identified by comparing DEGs with p53 knockdown (Figure 2B and Figure S2B). The analysis of the p53 binding sites in the ChIP peaks revealed that most of these genes were directly regulated by the p53, defined as p53 targets (Table S3). Additionally, the expression of most of the p53 targets correlated positively with the p53 levels, suggesting that p53 acts as an activator in HPV-infected cells, consistent with other studies showing p53 binding is associated with transcriptional activation [32,33].

However, the p53 targets differed significantly between the HPV18 and HPV16 cells. Similar results were obtained with varying log_2_ fold changes and Q-value thresholds. Only eight common p53 targets were identified in both the HPV16 and HPV18 cells, including AREG [34], BBC3 [35], BTG2 [36], CDKN1A [18], INPP5D [37], MDM2 [24], PADI4 [38], and SCN2A. Although genome-wide p53 binding is generally consistent across conditions [20], p53 binding productivity can be influenced by other factors nearby or interacting directly with p53 [39,40,41]. Consequently, p53 cofactors may vary between HPV16 and HPV18 cells. HPV16 is more carcinogenic than HPV18, with HPV16 accounting for 50% of cervical cancers and HPV18 for 15% [42]. Additionally, HPV16 infections predominantly lead to squamous cell carcinomas, while HPV18 infections often result in adenocarcinomas [43]. Future research should explore whether these distinct p53 targets contribute to the prevalence of HPV16 and HPV18 in cervical cancer. Additionally, the specific p53 targets involved in metabolism in HPV16 cells, such as CEL and XDH, warrant further investigation (Figure 5A and Figure S5).

The SCN2A gene encodes the voltage-gated sodium channel protein Nav1.2, which is crucial for action potential initiation and propagation in the central nervous system [44]. In this study, SCN2A was identified as a new p53 target in HPV-infected cells. A confirmed p53 binding site was located near the transcription start site of the RNA isoform NM_021007.3, associated with both a p53 ChIP peak and an active H3K4Me3 mark. Thus, this SCN2A isoform’s transcription was specifically activated by p53 in E6-silenced HPV cells. Further cell viability and apoptosis tests indicated that the SCN2A played a role in the p53-induced cell arrest but not in the apoptosis.

In summary, our genome-wide analysis characterized common and specific p53 targets under HPV conditions, with SCN2A identified as a novel p53 target involved in p53 pathways. Our study suggests that targeting SCN2A and other p53 targets could be a promising strategy for preventing HPV-related carcinogenesis.

## Data Availability

The data that support the findings of this study are available from the corresponding author, H.L., upon reasonable request.

## Supplementary Materials

The following supporting information can be downloaded at: https://www.mdpi.com/article/10.3390/v16111725/s1.
