# Supplementary material for: Genome-Wide Analysis of p53 Targets Reveals SCN2A as a Novel Player in p53-Induced Cell Arrest in HPV-Positive Cells
Source: Viruses. 2024 Oct 31;16(11):1725. doi: 10.3390/v16111725 (PMC11598893; doi:10.3390/v16111725)
Supplement: Supplementary file 1 [file viruses-16-01725-s001.zip › viruses-3271821-Figures.pdf]

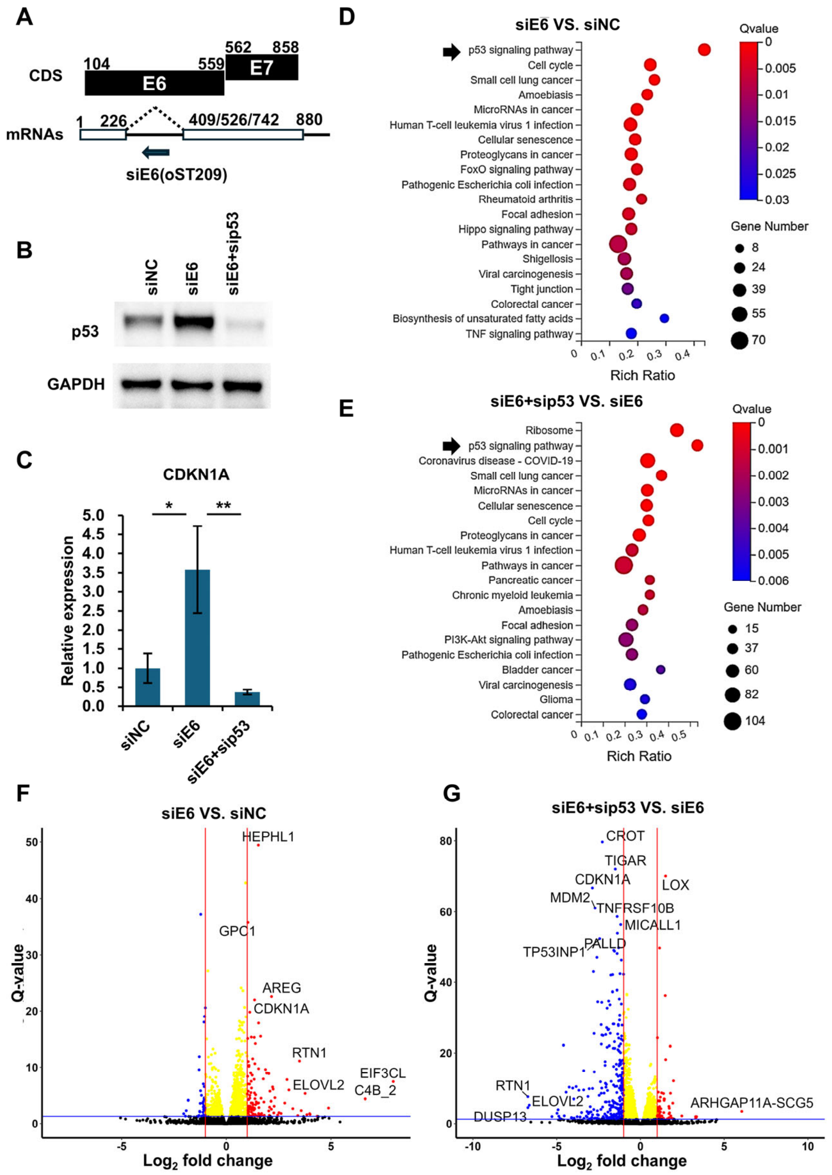

Figure S1. The regulated p53 pathway, by E6 knockdown, was verified in the HPV16 positive cell line (CaSki). (**A**) Intron-specific siRNA (siE6) similar to that used in Figure 1 targeting HPV16 RNA transcript. (**B**,**C**) p53 and CDKN1A expressions were examined by Western blotting (**B**) and qPCR analysis (**C**). *, $p < 0.05$; **, $p < 0.01$. (**D**,**E**) Top 20 enriched pathways in siE6 vs. siNC groups (**D**) and siE6 + sip53 vs. siE6 groups (**E**). p53 signaling pathways are highlighted by black arrows. (**F**,**G**) Volcano plots of DEGs used for pathway analysis.

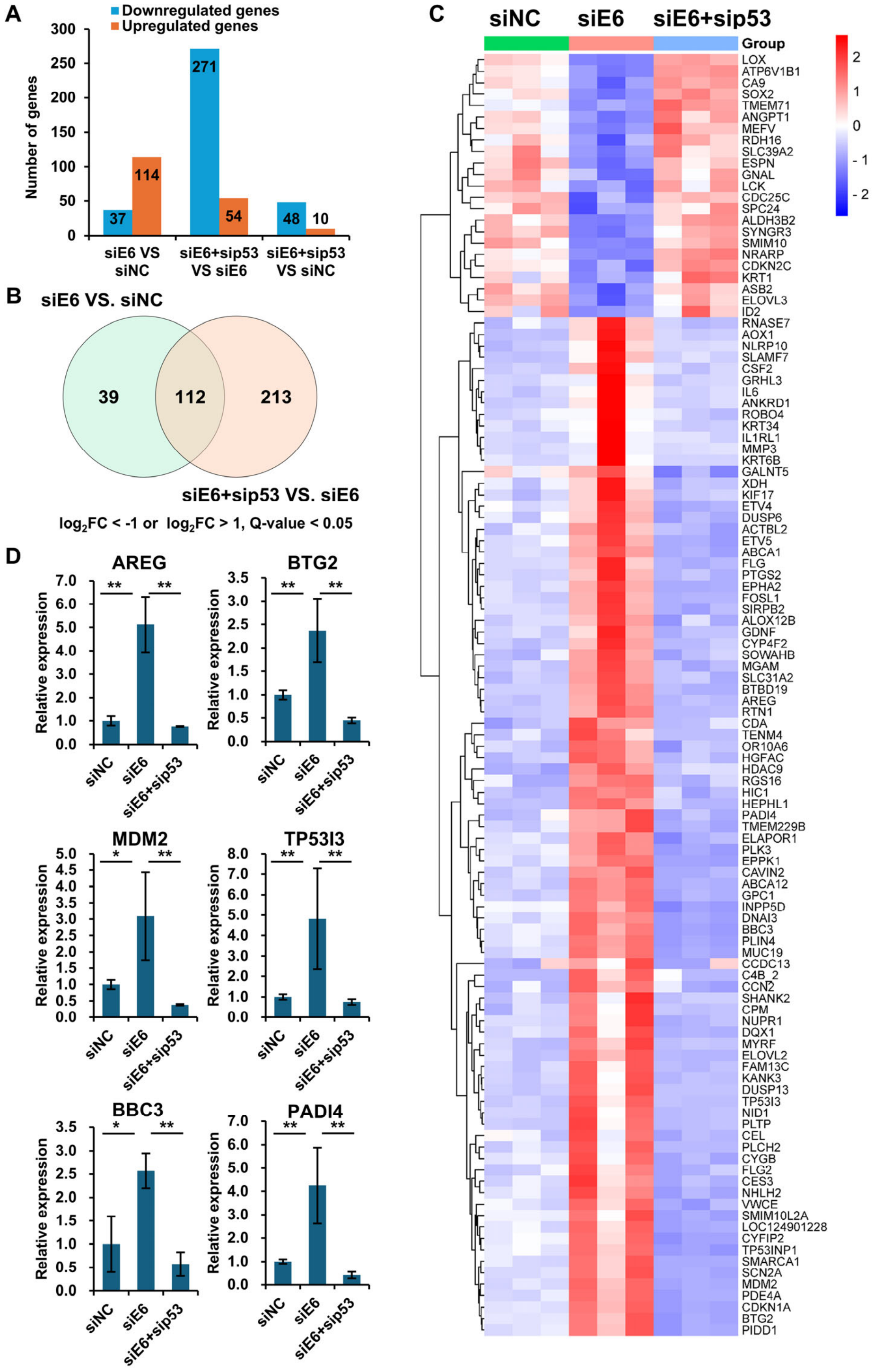

Figure S2. p53 also functions as an activator in the HPV16 positive cell line (CaSki). (**A**–**C**) Number of upregulated and downregulated genes (**A**) and overlapping DEGs visualized by Venn diagram (**B**) and heatmap (**C**). (**D**) Validation of expressions of selected DEGs. *, $p < 0.05$; **, $p < 0.01$.

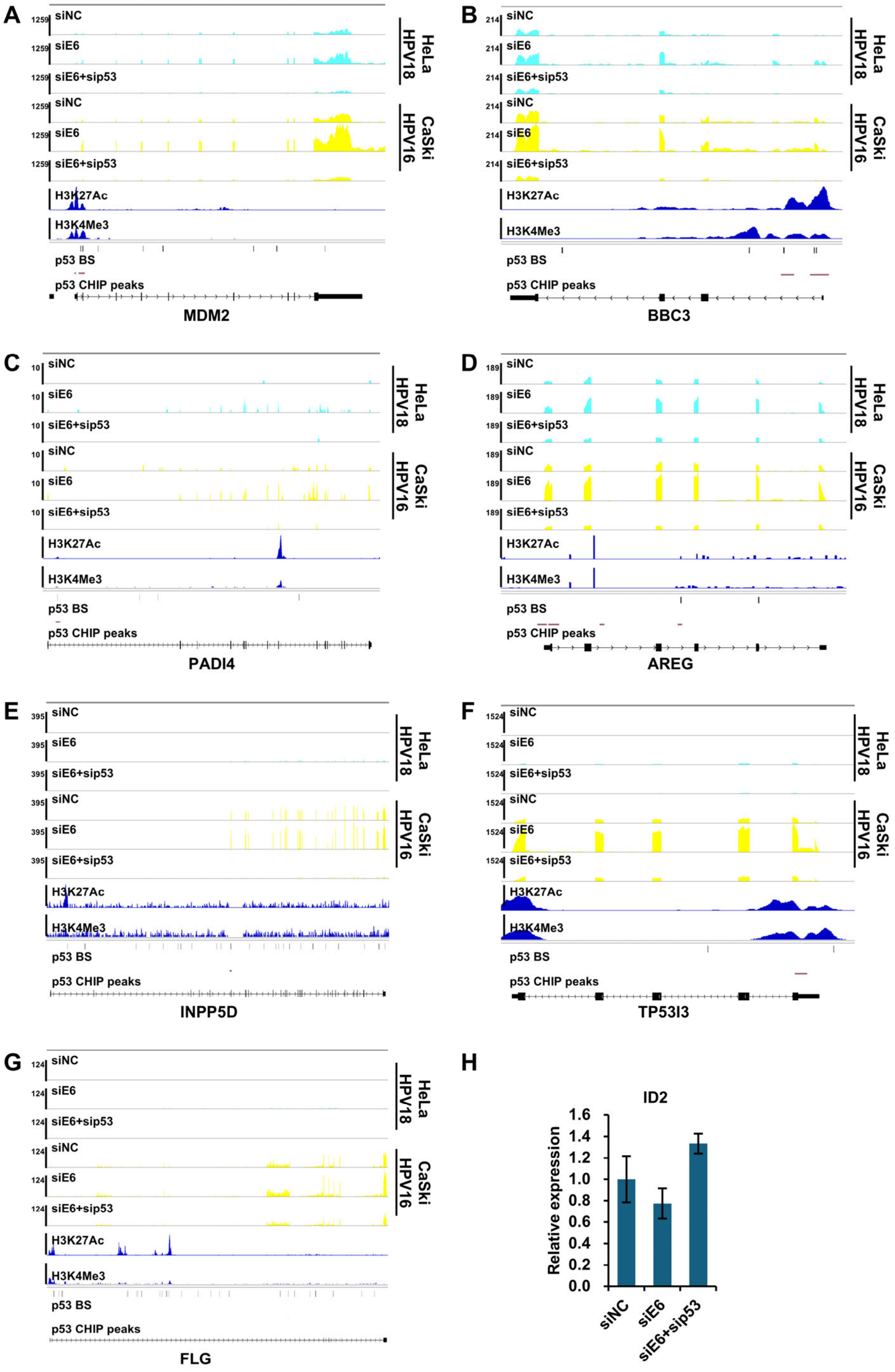

Figure S3. (**A**–**G**) Visualization of expressions, H3K27Ac mark, H3K4Me3 mark, p53 binding sites, and p53 CHIP peaks of other common p53 target genes in both HPV18 and HPV16 positive cell lines. (**H**) Validation of ID2 expression in siNC-, siE6-, or siE6 + sip53-transfected HPV18 cells by qPCR.

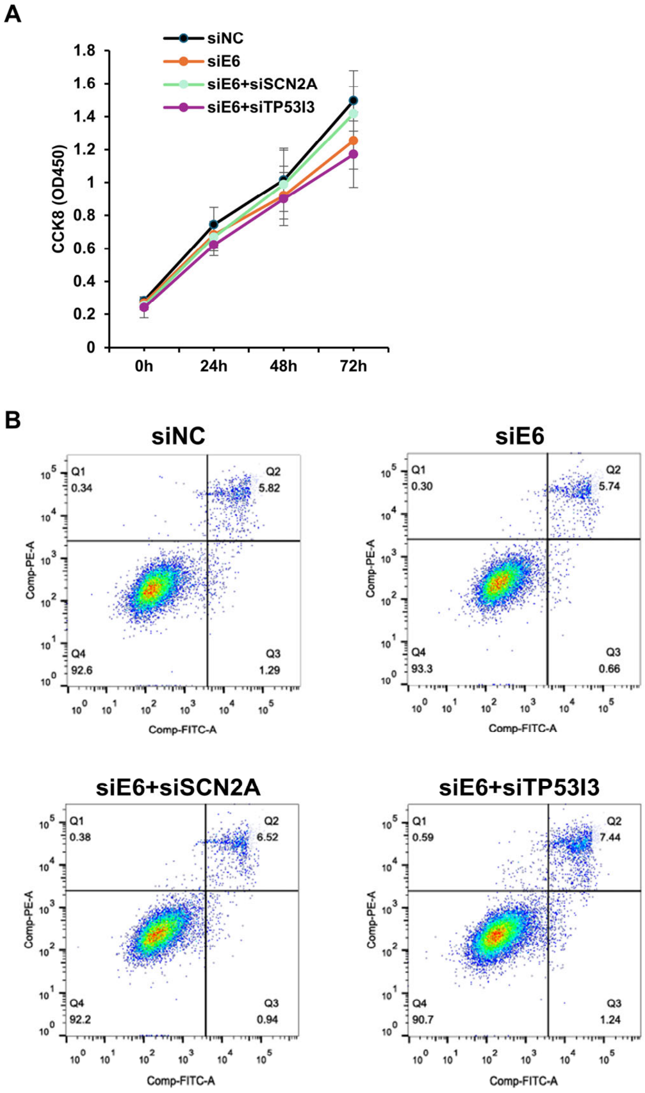

Figure S4. (**A**) The effects of E6, E6 + SCN2A, or E6 + TP53I3 knockdown on HeLa cell proliferation as described in Figure 4D, with each point representing an average of six replicates. (**B**) The effects of E6, E6 + SCN2A, or E6 + TP53I3 knockdown on cell apoptosis, measured by a flow cytometric analysis of FITC Annexin V staining at 72 h post-transfection with the indicated siRNAs**.**

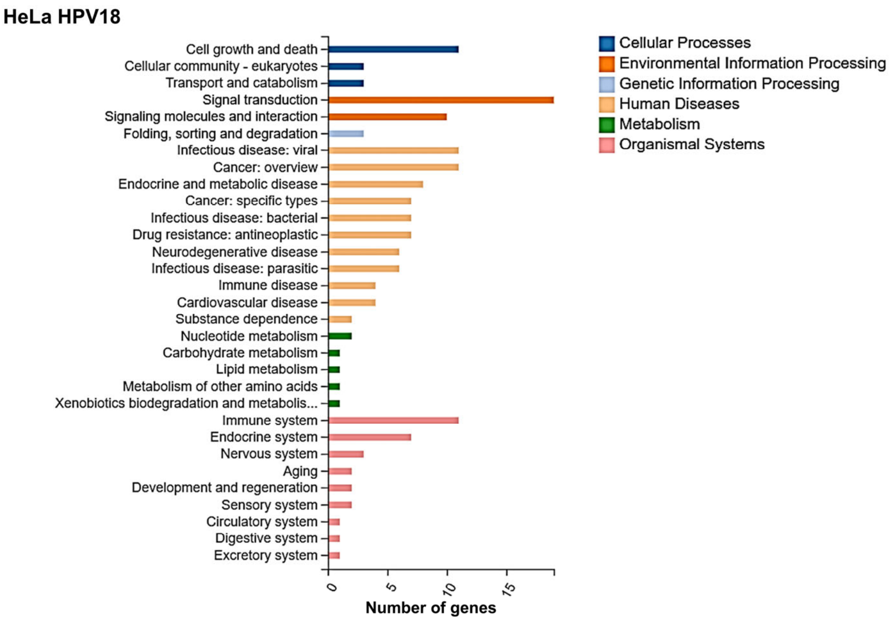

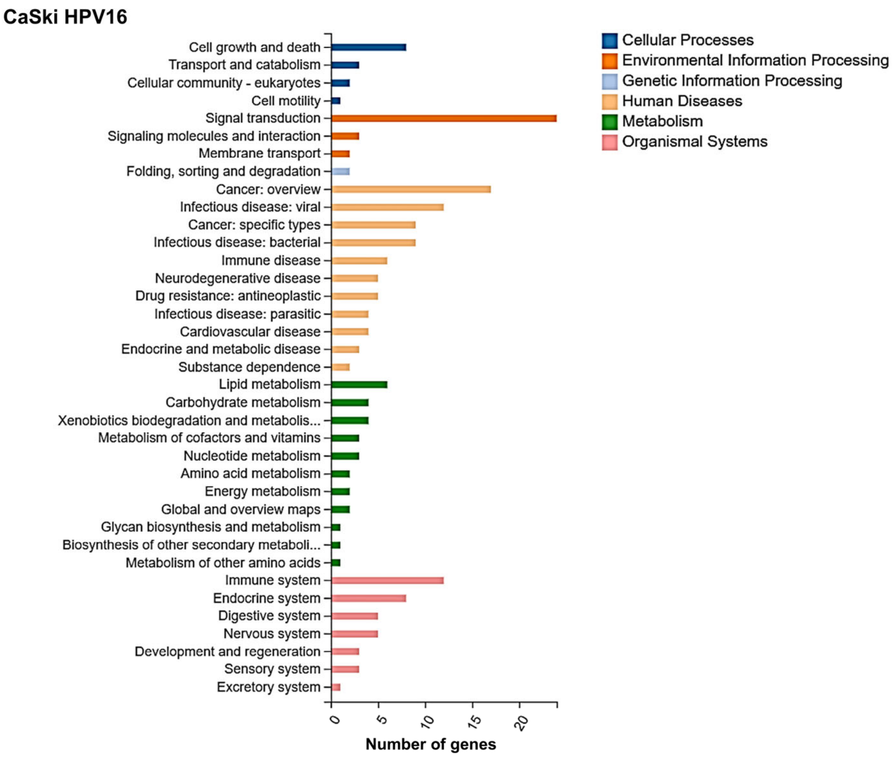

Figure S5. Enriched pathways in the HPV18 and HPV16 positive cell lines are categorized based on the KEGG PATHWAY database.
